# Evaluation of the efficacy, safety, and side effects of secukinumab in patients with moderate-to-severe psoriasis: real-world data from a retrospective multicenter study^☆^

**DOI:** 10.1016/j.abd.2021.11.002

**Published:** 2022-07-15

**Authors:** Ilteris Oguz Topal, Sevim Baysak, İlknur Kıvanç Altunay, Asude Kara Polat, Eylem Emel Arıkan, Ezgi Özkur, Sema Aytekin, Bilal Dogan, Tuğba Özkök Akbulut, Filiz Topaloğlu Demir, Ayşe Serap Karadağ

**Affiliations:** a Department of Dermatology and Venereology, University of Health Sciences, Prof. Dr. Cemil Tascioglu City Hospital, Istanbul, Turkey; b Department of Dermatology and Venereology, University of Health Sciences, Sultan II. Abdulhamid Han Training and Research Hospital, Istanbul, Turkey; c Department of Dermatology and Venereology, University of Health Sciences, Şişli Hamidiye Etfal Training and Research Hospital, Istanbul, Turkey; d Department of Dermatology and Venereology, University of Health Sciences, Istanbul Training and Research Hospital, Istanbul, Turkey; e Department of Dermatology and Venereology, Fatih Sultan Mehmet Training and Research Hospital, Istanbul, Turkey; f Department of Dermatology and Venereology, University of Health Sciences, Haydarpaşa Numune Training and Research Hospital, Istanbul, Turkey; g Department of Dermatology and Venereology, University of Health Sciences, Haseki Training and Research Hospital, Istanbul, Turkey; h Department of Dermatology and Venereology, Istanbul Medipol University, Faculty of Medicine, Istanbul, Turkey; i Department of Dermatology and Venereology, Memorial Atasehir Hospital, Turkey

**Keywords:** Efficacy, Psoriasis, Safety

## Abstract

**Background:**

Clinical studies have demonstrated that IL-17A inhibition with secukinumab is effective for clearing the skin of patients with psoriasis and has a favorable safety profile.

**Objective:**

The authors aim to determine whether secukinumab is effective and safe for the treatment of moderate-to-severe chronic psoriasis based on clinical experience with this drug.

**Method:**

The authors conducted a multicenter retrospective study in nine referral centers and included patients with psoriasis who had received secukinumab between March 2018 to November 2020. Data on demographic characteristics, Psoriasis Area and Severity Index (PASI) scores, and previous treatments were collected from medical records. Patients were evaluated at 12, 24, and 52 weeks with respect to response to treatment and side effects.

**Results:**

In total, 229 patients were recruited for the study. A PASI score improvement of ≥90 points over the baseline was achieved by 79%, 69.8%, and 49.3% of patients at weeks 12, 24, and 52, respectively. The most common adverse events were*Candida* infections and fatigue. In total, 74 (32%) patients discontinued treatment by week 52, including due to adverse events, or secondary ineffectiveness.

**Study limitations:**

Retrospective design.

**Conclusions:**

These findings suggest that secukinumab therapy is reasonably effective in patients with moderate-to-severe psoriasis. Comorbidities and time length of the disease can affect the response to treatment. The rates of adverse events were high in this patient population.

## Introduction

Psoriasis is an inflammatory, immune-mediated, systemic, chronic disease.^1^, ^2^ Epidemiologic studies from around the world have estimated the prevalence of psoriasis to be 0.6%–4.8%.^3^ In Turkey, a prevalence of 0.7%–1.8% has been reported in clinical studies.^4^, ^5^

Recently, the use of cytokine-targeted therapies has increased for the treatment of moderate-to-severe psoriasis. Secukinumab is a human monoclonal IgG1 k antibody that was developed to block the actions of IL-17A.^6^, ^7^, ^8^, ^9^ In 2015, an anti-IL-17 was approved for the first time, for the treatment of moderate-to-severe psoriasis and psoriatic arthritis in adult patients.^10^ Secukinumab has demonstrated high efficacy for the treatment of moderate-to-severe psoriasis and psoriatic arthritis, with a rapid onset of action, sustained response, and favorable safety profile.^11^, ^12^ Data from the ERASURE and FIXTURE studies revealed that a 300 mg subcutaneous (s.c.) dose achieves a peak effect at week 16, with efficacy sustained over 52 weeks of treatment.^13^ Regarding safety, IL-17A inhibitors are associated with various side effects and an increased incidence of candidal infections.^11^, ^13^ To date, there is a scarcity of literature on real-world data in terms of the efficacy and safety of secukinumab. In this study, the authors analyzed the efficacy, safety, and side effects of secukinumab in patients with moderate-to-severe psoriasis.

## Methods

### Patients and setting

Patients aged ≥18 years, treated with secukinumab for psoriasis at nine dermatology centers between March 2018 and November 2020, were included in the present study. The patients who did not receive secukinumab therapy and those who aged <18 years were excluded. The data were analyzed retrospectively. The sociodemographic characteristics of the patients (age, sex, Body Mass Index [BMI], comorbidities, and smoking and alcohol use) and disease-related features (previous conventional treatments [if any], previous biological agents, disease duration, and Psoriasis Area and Severity Index [PASI] score before initiating secukinumab) were collected from medical records. Secukinumab was administered according to a standard dosing regimen (300 mg s.c. once weekly for 5-weeks, and once a month thereafter). The response to secukinumab treatment was determined based on the PASI score at 12, 24, and 52 weeks. Treatment efficacy was indicated by the PASI 50, PASI 75, PASI 90, and PASI 100 response rates.

When secukinumab was discontinued, the reason for discontinuation (lack of treatment efficacy, adverse events, financial problems, or other causes) was noted. Adverse events such as infections, malignancy, and neutropenia were also recorded. If candida infection developed, clinical features such as the site of occurrence, the extent of involvement, and whether systemic therapy was needed were evaluated.

The study protocol was approved by the local Ethics Committee (Number: 439, Date: 17/11/2020) and all patients provided informed consent prior to participation.

### Statistical analysis

NCSS software (LLC, Kaysville, UT, USA) was used for the statistical analyses.

In addition to descriptive statistics (mean, standard deviation, median, frequency, and ratio), the Shapiro-Wilk test and box plots were used to normality of the data distribution. The groups were compared using the Chi-Square test, Mann-Whitney *U* test, Fisher’s exact test, and Spearman’s correlation analysis; p < 0.05 was accepted as statistically significant. The evaluations of efficacy were analyzed “as observed”, and patients who were not eligible for evaluation were not included in the analysis. Drug survival was analyzed using the Kaplan-Meier method while differences between groups were detected with the log-rank-test.

## Results

### Demographic features of patients

In total, 229 patients (139 [60.7%] males and 90 [39.3%] females) were included in the study. The mean age of the patients was 19 ± 78 years. The mean disease duration was 215.4 ± 129.4 months. Of the patients, 71 (31%) had a BMI ≥ 30 kg/m². Psoriatic arthritis was present in 78 (34.1%) patients. Smoking was reported by 93 (40.6%) patients. Comorbidities were present in 104 patients (45.41%); the most common were hypertension (17.9%), hyperlipidemia (12.23%), and diabetes mellitus (11.35%). The demographic characteristics of the patients are shown in Table 1.

**Table 1:** Demographic characteristics of the patients.

Characteristic	All patients (n = 229)
Age (years)	19 ± 78
Males, n (%)	139 (60.7)
Mean BMI (kg/m²)	28.3 ± 5.4
BMI ≥30 kg/m² n (%)	71 (31)
Smoking, n (%)	93 (40.6)
Alcohol use, n (%)	29 (12.7)
Psoriatic arthritis, n (%)	78 (34.1)
Psoriasis duration, months	215.4 ± 129.4
PASI score at baseline	12 ± 42.5
Comorbidities n (%)	
Hypertension	41 (17.9)
Hyperlipidemia	28 (12.23)
Diabetes mellitus	26 (11.35)
Asthma	15 (6.55)
Cardiac disease	7 (3.06)
Previous systemic treatments, n (%)	
Methotrexate	207 (90.39)
Acitretin	121 (52.84)
Cyclosporine	69 (30.13)
Phototherapy	83 (36.24)
Bio-naive patients	111 (48.4)
Bio-switched patients	118 (51.5)
Previous biological therapy, n (%)	
Adalimumab	78 (34.06)
Etanercept	38 (16.59)
Ustekinumab	38 (16.59)
Infliximab	18 (7.86)
Certolizumab	1 (0.44)
Golimumab	1 (0.44)

While 118 of 229 (51.5%) patients were not naive to biologics, 111 (48.4%) patients had not previously received biological treatment (bio-naive) (Table 1). The previous treatments are listed in Table 1.

### Effectiveness

**Figure 1:**
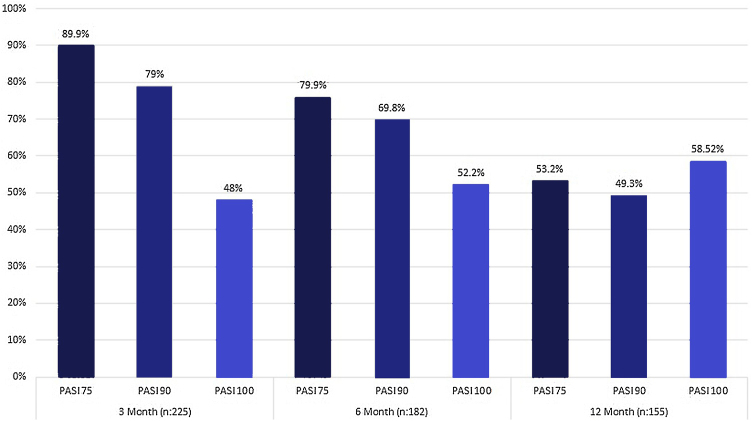
“As-observed” response rate under secukinumab 300 mg.

The mean PASI score of patients before treatment was 12 ± 42.5. At week 12 of treatment, 89.9% of patients achieved PASI 75, 79% achieved PASI 90, and 48% achieved PASI 100. The PASI 75, 90, and 100 response rates at 24 and 52 weeks are summarized in Fig. 1. At 12, 24, and 52 weeks, there was no significant difference in the improvement of PASI scores between bio-naive and “bio-switched” patients (p > 0.5). While 182 (79.4%) patients reached week 24 of secukinumab treatment, 155 (68%) reached week 52 (Fig. 1). No significant difference in PASI 75, 90, and 100 response rates after treatment was found between the BMI ≥ 30 kg/m² and a BMI < 30 kg/m² groups (p > 0.5). Also, no significant difference in the PASI 75, PASI 90 or PASI 100 response rate was found between patients with and without arthritis (p > 0.5).

**Figure 2:**
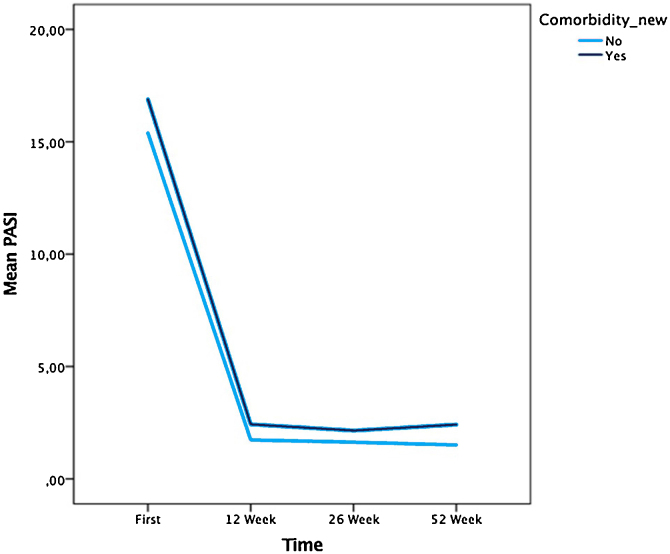
The mean PASI score according to comorbidities.

The mean PASI score was lower in patients without comorbidities at 12-weeks than in those with comorbidity, according to the Mann-Whitney *U* test (p = 0.034) (Fig. 2).

There was a significant association between the PASI score and disease duration at 24-weeks. Shorter disease duration was associated with a greater PASI reduction according to Spearman’s correlation analysis (p = 0.016).

Age, sex, and the number of biological drugs used before secukinumab were not found to have a significant impact on the therapeutic response to secukinumab (p > 0.5).

### Safety and side effects

Side effects were observed in 135 (58.9%) patients using secukinumab. The most common adverse event was candidal infection (10.4%), followed by fatigue (7.42%) and nasopharyngitis (6.99%) (Table 2). Of the 229 patients, 23 (10.04%) had mucocutaneous candida infections, commonly seen as vulvovaginal and intertriginous candidiasis (Table 2). Of the 23 patients, 14 were treated with topical antifungal creams. Only five patients were treated with systemic antifungal therapy, of whom four (1.75%) discontinued the secukinumab treatment. Of the 74 (32%) patients who discontinued secukinumab, the reasons for discontinuation included various adverse events in 24 (10.48%), secondary ineffectiveness in 20 (8.73%), non-adherence to treatment in 12 (5.24%), patient request in 12 (5.24%), and primary ineffectiveness in 2 (0.87%) (Table 3).

**Table 2:** The most common adverse events in patients treated with secukinumab.

Adverse event	n (%)
Candida infections	23 (10.4)
Vaginal	9 (3.93)
Intertriginous	9 (3.93)
Oropharyngeal	4 (1.75)
Onychomycosis	3 (1.31)
Erosio interdigitalis blastomycetica	3 (1.31)
Fatigue	17 (7.42)
Nasopharyngitis	16 (6.99)
Paradoxical arthritis	13 (5.68)
Bronchitis	4 (1.75)
Arthralgia	4 (1.75)
Pruritus	3 (1.31)
Increase in weight	3 (1.31)

**Table 3:** Reasons for discontinuation in patients treated with secukinumab.

Reasons for discontinuation	n = 74 (32%)
Adverse events	24 (10.48)
Secondary ineffectiveness	20 (8.73)
Non-adherence to treatment	12 (5.24)
Patient request	12 (5.24)
Primary ineffectiveness	2 (0.87)
Others	2 (0.87)
Pregnancy	1 (0.44)
Financial problems	1 (0.44)

### Drug survival

**Figure 3:**
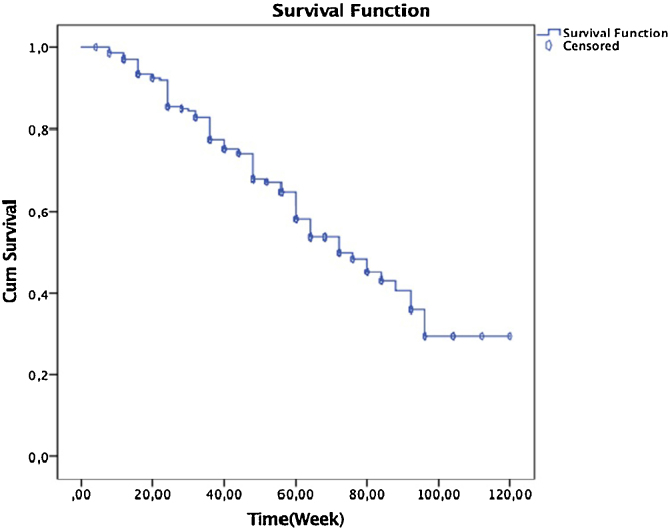
Drug survival of secukinumab 120 week (30% [95% CI 68.2‒81], MST: 74.6-week), week (68% [95% CI 43.7‒47], MST: 45.4-week).

The mean duration of follow-up for patient was forty seven weeks (4 weeks ‒ 120 weeks). Fig. 3 demonstrates the Kaplan-Meier drug survival curve in this patient cohort. Drug survival at week 52 was 68% (155 patients) and at week 120 was 30% (68 patients) (Fig. 3).

The authors also carried out a sub-analysis considering either adverse events or secondary ineffectiveness as the cause of discontinuation. For adverse events, there was no significant difference in persistence to therapy between those who continued treatment and did not continue the group. Persistence to therapy was 62% (Median Survival Time = 52-week), 10% (Median Survival Time = 45-week) for those patients, respectively.

With respect to secondary ineffectiveness, a comparison of the drug survival rate between those who continued treatment and did not continue group showed no significant differences.

According to Kaplan-Meier analysis gender, age (<50-years or >50-years), BMI (<30 or ≥30), presence of psoriatic arthritis or comorbidities, and previously received biological treatment did not seem too significant effect overall secukinumab survival.

## Discussion

Secukinumab is a biological drug used for the treatment of psoriasis and acts by inhibiting IL-17A.^14^ Various real-world studies have reported on the efficacy and safety of secukinumab in psoriasis (Table 4). In a recent prospective multicenter study including 158 patients, the proportions of patients achieving PASI 75 at weeks 12, 24, and 52 were found to be 83.5%, 89%, and 78.5%, respectively; the proportions achieving PASI 90 at the same time points were 62%, 64.6%, and 63.2%.^15^ The authors found PASI 75 and PASI 90 responses of 79.9% and 69.8% at week 24, and 53.2% and 49.3% at week 52, respectively. The proportions achieving PASI 100 at the same points were 52.2% and 58.52%, respectively.

**Table 4:** Real-world studies on the effectiveness of secukinumab.

Study	Design	Number of patients	Baseline characteristics				Effectiveness			Side effects (%)
			Age (years, mean)	Male (%)	Psoriatic arthritis (%)	Comorbidities (%)	12-week PASI 75/ 90/ 100 (%)	24-week PASI 75/ 90/ 100 (%)	52-week PASI 75/ 90/ 100 (%)	
Schwensen et al. (2017)	Retrospective	69	48 (40.5‒57.5)	‒	43.5	‒	52.9/ 35.3	‒	‒	26.1
Momose et al. (2017)	Retrospective	83	57.3 ± 15.2	68.7	14.4	‒	80/ 64/ 53	77/ 65/ 51	76/ 58/ 43	‒
Galluzzo et al. (2018)	Retrospective	107	47.5 ± 12.8	75	14.9	51.3	80/ 67.5/ 55	76.8/ 71/ 58	92/ 81.6/ 78.9	9.3
Notario et al. (2019)	Retrospective	136	49 ± 12.7	71.3	33.1	‒	67.2/ 53.7 (16 week)	‒	69/ 46	‒
Ortiz-Salvador et al. (2019)	Prospective	158	28 ± 17.7	57	34.8	‒	83.5/ 62	89/ 64.6	78.5/ 63.2	17.7
Romboti et al. (2019)	Retrospective	83	48.0 (21–76)	51	43.9	‒	83.8/ 70/ 46.3 (16 week)	‒	92/ 86/ 40	7.2
Ger et al. (2019)	Retrospective	118	48.0 ± 13.8	74.5	39.8	‒	64/ 28	63/ 32	53.7/ 27.8 (48 week)	61
Carpentiari et al. (2020)	Retrospective	120	49.8 ± 13.5	64.2	38.3	47.5	‒	‒	‒	23.3
Huang et al. (2020)	Retrospective	81	40.1 ± 11.1	67.9	19.8	‒	91.1/ 73/ 38.3 (16 week)	‒	‒	42
Zhao et al. (2021)	Retrospective	106	39.6 ± 12.2	64.1	14.1	79.2	93.2/ 81.4/ 76.2	91.5/ 86.4/ 79.9	‒	47.2
Current study	Retrospective	229	19 ± 78	60.7	34.1	45.41	89.9/ 79/ 48	79.9/ 69.8/ 52.2	53.2/ 49.3/ 58.52	58.9

Georgakopoulos et al. suggested that fewer psoriasis patients in real-world clinical practice maintain efficacious outcomes at week 52 than those enrolled in randomized-controlled studies.^16^ Although PASI 75/90 response decreased gradually over 52-weeks of secukinumab, the PASI 100 response rate was higher in the present study’s cohort.

In another study, 69 patients with psoriasis who started treatment with secukinumab primarily due to failure of anti-TNF and/or anti-IL12-23 treatment were evaluated. At week 12, 66.7% of the patients still on secukinumab had experienced at least a 50% reduction in PASI (PASI 50) from baseline.^17^ In the present study, 225 (98%) patients were still receiving treatment at week 12 and approximately 50% of them achieved PASI 100.

In Notario et al.’s study, the percentages of patients with a BMI ≥ 30 kg/m² who achieved PASI 75 and PASI 90 responses were significantly lower than those with a BMI < 30 kg/m^2^.^18^ In another study, an analysis of all patients receiving 300 mg secukinumab for 12 weeks showed that the proportion of PASI 75 responders was lower among those with a BMI > 25 kg/m^2^ than in those with a BMI < 25 kg/m^2^.^19^ In contrast, Schwensen et al. reported that baseline PASI scores were not different according to obesity status (and where BMI > 25 kg/m^2^ is considered obese).^17^ Similar to Schwensen et al., the authors did not detect an association between BMI and PASI response.

Galluzzo et al. showed that younger patients responded to treatment more quickly: the rates of PASI 75, PASI 90, and PASI 100 were higher in those patients at week 4. Other parameters, such as sex and the PASI score at baseline, were not associated with the PASI 75, PASI 90, and PASI 100 response rates.^20^ Similarly, Huang et al. observed that young age was associated with a better clinical response to secukinumab.^21^ There was a significant association between the PASI response and disease duration in the present study. Shorter disease duration was associated with lower PASI scores at week 24 (p = 0.016), but there were no such associations with age or sex. In another study, PASI 75 response was found lower in patients with psoriatic arthritis when compared to patients without psoriatic arthritis.^22^ Ortiz-Salvador et al. showed that PASI 75 and PASI 90 rates were not associated with the baseline PASI score, age, sex, smoking status, or presence of psoriatic arthritis or dyslipidemia.^15^ The degree of improvement in the PASI score had no association with psoriatic arthritis in the present study’s cohort, but the mean PASI score was lower in patients without comorbidities at week 12 (p = 0.034).

Galluzzo et al. enrolled 107 patients (75% males) with psoriasis, with a mean age of 47.5 years, in their retrospective study. Approximately 51.3% of the patients had comorbidities; the most frequent were obesity (23.4%), hypertension (15%), hyperlipidemia (13.1%), and type 2 diabetes (10.3%).^20^ Similarly, in the present study, the most frequently observed comorbidities were obesity (31%), hypertension (17.9%), hyperlipidemia (12.23%), and diabetes mellitus (11.35%). Galluzzo et al. also showed that PASI 75, PASI 90, and PASI 100 were achieved more frequently by treatment-naive patients at weeks 12 and 24. In total, 55 (51.4%) patients had previously been treated with other biologic therapies, and 52 (48.6%) were naive to biologics.^20^ Similarly, in another study, prior exposure to ≥1 biological therapy was associated with a diminished therapeutic response to secukinumab at week 16.^18^ In Ger’s study, the response rates of PASI 50, and PASI 75 at weeks 12 and 24 in patients without prior biologic failure were also significantly greater than those with prior biologic failure.^23^ In the present study, while 111 (48.4%) patients were bio-naive, 118 (51.5%) were bio-switched. With respect to the PASI response, there was no significant difference between the bio-naive and bio-switched patients (p > 0.5). In another study, no significant difference in efficacy was observed between bio-naive and bio-switched patients.^24^

Carpentieri et al. divided their secukinumab patients into the bio-naive, history of the inefficacy of one biological agent, and unresponsive to ≥2 biological agent groups. At the end of the study, they reported that PASI scores had declined at 3 and 12-months in all groups.^25^ The authors also analyzed patients who had received one or more than one biological agents. After treatment, the PASI score had declined in both groups at 12, 24, and 52 weeks.

Carpentieri et al. found that 18 of 120 patients (15%) discontinued secukinumab (8 due to adverse events and 10 due to lack of efficacy).^25^ Notario et al. reported a discontinuation rate of 21.3% (29/136). The reasons cited for discontinuation included serious adverse events, lack of efficacy, loss of follow-up, pregnancy, and major surgery.^18^ In another study, the patients who discontinued and pursued treatment received secukinumab for a median of 25.5 and 99.9 weeks, respectively. Out of 91 patients, 22 (24.2%) discontinued secukinumab due to loss of efficacy (14 patients, 15%), adverse events (5 patients, 5.4%), desire for pregnancy (2 patients), and loss to follow-up (1 patient).^26^ In the study of Ortiz-Salvador et al., 27 (17.1%) patients discontinued treatment due to a lack of efficacy (8 patients), loss of efficacy (15 patients), or loss of follow-up (4 patients).^15^

In the present study, 155 (68%) patients reached treatment week 52, and 74 (32%) discontinued treatment. Reasons for discontinuation included various adverse events (10.48%), secondary ineffectiveness (8.73%), non-adherence to treatment (5.24%), and patient request (5.24%), among other reasons. The most common adverse events related to discontinuation were fatigue, nasopharyngitis, and candidal infections.

In real-world studies, the adverse events rate for secukinumab ranged from 7.2% to 61%.^22^, ^23^ Adverse events reported by Zhao et al. included nasopharyngitis, superficial skin bacterial infections, itching, urticaria, and eczema.^27^ Ortiz-Salvador et al. reported adverse events in 28 patients (17.7%), the most frequent of which were headache (5.7%), nasopharyngitis (5.7%), and hypertension (3.8%).^15^ In the SIGNATURE study, which investigated the efficacy of secukinumab in patients for whom TNF-α inhibitor therapy had failed, treatment-emergent adverse events occurred in 83.7% of cases; however, most of the events were mild or moderate in severity.^28^

Recently, the authors analyzed the adverse effects of the biological agents used in the treatment of psoriasis. The rate of adverse effects was 67.4% in patients using anti-TNF-α agents, and 55.3% in those using IL inhibitors (ustekinumab and secukinumab). However, the rates of serious adverse effects were similar (4.8% in the anti-TNF-α group and 3% in the IL inhibitors), (but less frequent in secukinumab [1.6%]).^29^

In the current study, there were no serious adverse events with secukinumab treatment for up to 52-weeks. The rate of adverse events was 58.9% (135 patients). The most commonly observed adverse effect was candidal infection (10.04%), followed by fatigue (7.42%).

The IL-17 pathway regulates immunity in candida infection, probably via upregulation of proinflammatory cytokines (IL-6 and neutrophil-recruiting chemokines) among other actions, and the use of IL-17 inhibitors have was associated with an increased risk of mucocutaneous candidiasis.^30^ The rate of candida infection with the use of secukinumab varies between 0.0% and 5.0%.^31^ In a Japanese study, candida infection was detected in only 1 of 52 patients with psoriasis and completely resolved within 14-days without discontinuation of treatment.^32^ However, in another study of 91 patients taking secukinumab, the treatment was stopped in 1 patient with oral candidiasis.^22^ In two other studies, the rates of candida infection were identical (3.7%).^15^, ^18^

A recent study reported candida infections in 16 patients with psoriasis treated with secukinumab 300 mg for 12 months. All patients were evaluated clinically, and swab samples and coculture for candida infection were obtained during therapy. Oral swabs were positive for candida Albicans in two patients and coproculture was positive in one patient. After 12 months of secukinumab therapy, all patients were negative for candida infection, although no antifungal therapy was prescribed, and no patient showed clinical signs of candida infection.^33^

In another study, candida infection was reported in 5.7% of patients. The most common forms of candidiasis were oral (3.6%), vulvovaginal (0.9%), genital (0.3%), and esophageal (0.6%).^34^

The authors observed candida infection in 23 patients (10.04%). This high rate may be related to the thoroughness of the examinations, particularly for candida. In most cases, candidiasis was vulvovaginal, intertriginous, or oropharyngeal. Topical treatment was sufficient for most patients; systemic therapy was used in only five patients. Four of those patients (1.75%) discontinued secukinumab treatment due to candidal infection; most had mucosal involvement.

Paradoxical reactions, such as palmoplantar psoriasis and inverse psoriasis, have been described in the literature in association with anti-TNF-α inhibitors, secukinumab, and ustekinumab. Paradoxical arthritis may be triggered by ustekinumab, but has rarely been reported in association with secukinumab.^24^, ^35^ Interestingly, paradoxical arthritis developed in 13 of the patients (5.68%) with psoriasis. The authors suspect that arthritis occurred as a result of changes in cytokine balance and previously used biological agents.

The limitations of this study include its retrospective design, which caused some difficulties with respect to data access. In addition, due to the small number of patients in some groups, analysis of minor differences was not possible.

## Conclusion

In this study, the PASI 90 and PASI 100 response rates were high in patients with moderate-to-severe psoriasis. Unlike other studies, the authors found that PASI scores declined more rapidly in patients with a short disease duration at 24-weeks, and lower mean PASI scores were achieved in patients without comorbidities at 12-weeks. These results show that factors such as the presence of comorbidities and disease duration can affect PASI responses in a negative way. As in other studies, adverse events such as fatigue and nasopharyngitis occurred in the studied cohort. However, the rates of candidal infection and paradoxical arthritis were higher in the present patient population compared to other real-world studies.

## Financial support

None declared.

## Authors' contributions

Ilteris Oguz Topal: Study conception and planning, preparation and writing of the manuscript, final approval of the final version of the manuscript.

Sevim Baysak: Data collection and/or processing, investigation.

Ilknur Kıvanc Altunay: Manuscript critical review, writing of the manuscript, analysis and data interpretation.

Asude Kara Polat: Data collection and/or processing, methodology.

Eylem Emel Arıkan: Data collection and/or processing.

Ezgi Özkur: Data collection and/or processing, literature review.

Sema Aytekin: Data collection and/or processing, literature review.

Bilal Dogan: Data collection and/or processing.

Tuğba Özkök Akbulut: Data collection and curation.

Filiz Topaloğlu Demir: Data collection and curation.

Ayse Serap Karadağ: Manuscript critical review, data collection.

## Conflicts of interest

None declared.

## References

[1] Stern R.S., Nijsten T., Feldman S.R., Margolis D.J., Rolstad T. (2004). Psoriasis is common, carries a substantial burden even when not extensive, and is associated with widespread treatment dissatisfaction. J Investig Dermatol Symp Proc..

[2] Menter A., Gottlieb A., Feldman S.R., Van Voorhees A.S., Leonardi C.L., Gordon K.B. (2008). Guidelines of care for the management of psoriasis and psoriatic arthritis: Section 1. Overview of psoriasis and guidelines of care for the treatment of psoriasis with biologics. J Am Acad Dermatol..

[3] Gudjonsson J.E., Elder J.T. (2007). Psoriasis: epidemiology. Clin Dermatol..

[4] Aykol C., Mevlitoğlu İ, Özdemir M., Ünal M. (2011). Evalution of Clinical and Sociodemograpic Features of Patients with Psoriasis in the Konya Region. Turk J Dermatol..

[5] Turan H., Acer E., Aliağaoğlu C., Uslu E., Albayrak H., Özşahin M. (2013). The Evaluation of the Sociodemografic and Clinical Features of Patients with Psoriasis. Turk J Dermatol..

[6] Roman M., Madkan V.K., Chiu M.W. (2015). Profile of secukinumab in the treatment of psoriasis: current perspectives. Ther Clin Risk Manag..

[7] Lønnberg A.S., Zachariae C., Skov L. (2014). Targeting of interleukin-17 in the treatment of psoriasis. Clin Cosmet Investig Dermatol..

[8] Gaffen S.L., Jain R., Garg A.V., Cua D.J. (2014). The IL-23-IL-17 immune axis: from mechanisms to therapeutic testing. Nat Rev Immunol..

[9] Patel D.D., Lee D.M., Kolbinger F., Antoni C. (2013). Effect of IL-17A blockade with secukinumab in autoimmune diseases. Ann Rheum Dis..

[10] Sanford M., Mackeage K. (2015). Secukinumab: first global approval. Drugs..

[11] Blauvelt A., Prinz J.C., Gottlieb A.B., Kingo K., Sofen H., Ruer-Mulard M. (2015). Secukinumab administration by pre-filled syringe: efficacy, safety, and usability results from a randomized controlled trial in psoriasis (FEATURE). The Br J Dermatol..

[12] Abrouk M., Gandy J., Nakamura M., Lee K., Brodsky M., Singh R. (2017). Secukinumab in the Treatment of Psoriasis and Psoriatic Arthritis: A Review of the Literature. Skin Therapy Lett..

[13] Armstrong A.W., Papp K., Kircik L. (2016). Secukinumab: Review of Clinical Evidence from the Pivotal Studies ERASURE, FIXTURE, and CLEAR. J Clin Aesthet Dermatol..

[14] Frieder J., Kivelevitch D., Menter A. (2018). Secukinumab: a review of the anti-IL-17A biologic for the treatment of psoriasis. Ther Adv Chronic Dis..

[15] Ortiz-Salvador J.M., Saneleuterio-Temporal M., Magdaleno-Tapial J., Velasco-Pastor M., Pujol-Marco C., Sahuquillo-Torralba A. (2019). A prospective multicenter study assessing effectiveness and safety of secukinumab in a real-life setting in 158 patients. J Am Acad Dermatol..

[16] Georgakopoulos J.R., Ighani A., Phung M., Yeung J. (2018). Drug survival of secukinumab in real-world plaque psoriasis patients: A 52-week, multicenter, retrospective study. J Am Acad Dermatol..

[17] Schwensen J.F., Clemmensen A., Sand C., Gniadecki R., Skov L., Zachariae C. (2017). Effectiveness and safety of secukinumab in 69 patients with moderate to severe plaque psoriasis: A retrospective multicenter study. Dermatol Ther..

[18] Notario J., Deza G., Vilarrasa E., Valentí F., Muñoz C., Mollet J. (2019). Treatment of patients with plaque psoriasis with secukinumab in a real-life setting: a 52-week, multicenter, retrospective study in Spain. J Dermatolog Treat..

[19] Wu N.L., Hsu C.J., Sun F.J., Tsai T.F. (2017). Efficacy and safety of secukinumab in Taiwanese patients with moderate to severe plaque psoriasis: Subanalysis from ERASURE phase III study. J Dermatol..

[20] Galluzzo M., Talamonti M., Simone C., D’Adamio S., Moretta G., Tambone S. (2018). Secukinumab in moderate-to-severe plaque psoriasis: a multi-center, retrospective, real-life study up to 52 weeks observation. Expert Opin Biol Ther..

[21] Huang H., Cai M.L., Hong X.J., Zheng L.J., Hu Z.L., Yuan T. (2020). Real-world data on the use of secukinumab as treatment for moderate-to-severe psoriasis in Chinese patients. Eur J Dermatol..

[22] Rompoti N., Katsimbri P., Kokkalis G., Boumpas D., Ikonomidis I., Theodoropoulos K. (2019). Real world data from the use of secukinumab in the treatment of moderate-to-severe psoriasis, including scalp and palmoplantar psoriasis: A 104-week clinical study. Dermatol Ther..

[23] Ger T.Y., Huang Y.H., Hui R.C., Tsai T.F., Chiu H.Y. (2019). Effectiveness and safety of secukinumab for psoriasis in real-world practice: analysis of subgroups stratified by prior biologic failure or reimbursement. Ther Adv Chronic Dis..

[24] Momose M., Asahina A., Umezawa Y., Nakagawa H. (2018). Long-term clinical efficacy and safety of secukinumab for Japanese patients with psoriasis: A single-center experience. J Dermatol..

[25] Carpentieri A., Mascia P., Fornaro M., Beylot-Barry M., Taieb A., Foti C. (2020). Effectiveness and safety of secukinumab in patients with moderate-severe psoriasis: A multicenter real-life study. Dermatol Ther..

[26] Ferrières L., Konstantinou M.P., Bulai Livideanu C., Hegazy S., Tauber M., Amelot F. (2019). Long-term continuation with secukinumab in psoriasis: association with patient profile and initial psoriasis clearance. Clin Exp Dermatol.

[27] Zhao Y., Cai L., Liu X.Y., Zhang H., Zhang J.Z. (2021). Efficacy and safety of secukinumab in Chinese patients with moderate-to-severe plaque psoriasis: a real-life cohort study. Chin Med J (Engl)..

[28] Warren R.B., JNWB Barker, Finlay A.Y., Burden A.D., Kirby B., Armendariz Y. (2020). Secukinumab for patients failing previous tumour necrosis factor-α inhibitor therapy: results of a randomized open-label study (SIGNATURE). Br J Dermatol..

[29] Demir F.T., Akbulut T.Ö, Altunay İK., Aytekin S., Topal İO., Polat A.S. (2020). Evaluation of the adverse effects of biological agents used in the treatment of psoriasis: A multicenter retrospective cohort study. Dermatol Ther..

[30] Hay R.J. (2017). Candida infections and interleukin-17 inhibitors used in dermatology. Br J Dermatol..

[31] Saunte D., Mrowietz U., Puig L., Zachariae C. (2017). Candida infections in psoriasis and psoriatic arthritis patients treated with IL-17 inhibitors and their practical management. Br J Dermatol..

[32] Okubo Y., Ohtsuki M., Morita A., Yamaguchi M., Shima T., Tani Y. (2019). Long-term efficacy and safety of secukinumab in Japanese patients with moderate to severe plaque psoriasis: 3-year results of a double-blind extension study. J Dermatol..

[33] Papini M., Natalini Y. (2018). Candida infections in psoriatic patients on anti-IL17 therapy: a case series. J Dermatolog Treat..

[34] Thaçi D., Blauvelt A., Reich K., Tsai T.F., Vanaclocha F., Kingo K. (2015). Secukinumab is superior to ustekinumab in clearing skin of subjects with moderate to severe plaque psoriasis: CLEAR, a randomized controlled trial. J Am Acad Dermatol..

[35] Čarija A., Ivić I., Marasović-Krstulović D., Puizina-Ivić N. (2015). Paradoxical psoriatic arthritis in a patient with psoriasis treated with ustekinumab. Rheumatology (Oxford).

